# Registered report: Oncometabolite 2-hydroxyglutarate is a competitive inhibitor of α-ketoglutarate-dependent dioxygenases

**DOI:** 10.7554/eLife.07420

**Published:** 2015-07-31

**Authors:** Brad Evans, Erin Griner, Reproducibility Project: Cancer Biology, Elizabeth Iorns

**Affiliations:** Science Exchange, Palo Alto, California; Mendeley, London, United Kingdom; Science Exchange, Palo Alto, California; Science Exchange, Palo Alto, California; Center for Open Science, Charlottesville, Virginia; 1 Proteomics and Mass Spectrometry Facility, Donald Danforth Plant Science Center, St. Louis, Missouri, United States; 2 University of Virginia, Charlottesville, Virginia, United States; Institut de Génétique et de Biologie Moléculaire et Cellulaire, France

**Keywords:** methodology, Reproducibility Project: Cancer Biology, IDH, 2-hydroxyglutarate, human

## Abstract

The Reproducibility Project: Cancer Biology seeks to address growing concerns about reproducibility in scientific research by conducting replications of selected experiments from a number of high-profile papers in the field of cancer biology. The papers, which were published between 2010 and 2012, were selected on the basis of citations and Altmetric scores (Errington et al., 2014). This Registered report describes the proposed replication plan of key experiments from ‘Oncometabolite 2-hydroxyglutarate is a competitive inhibitor of α-ketoglutarate-dependent dioxygenases’ by Xu and colleagues, published in *Cancer Cell* in 2011 (Xu et al., 2011). The key experiments being replicated include Supplemental Figure 3I, which demonstrates that transfection with mutant forms of IDH1 increases levels of 2-hydroxyglutarate (2-HG), Figures 3A and 8A, which demonstrate changes in histone methylation after treatment with 2-HG, and Figures 3D and 7B, which show that mutant IDH1 can effect the same changes as treatment with excess 2-HG. The Reproducibility Project: Cancer Biology is a collaboration between the Center for Open Science and Science Exchange, and the results of the replications will be published by *eLife*.

**DOI:** http://dx.doi.org/10.7554/eLife.07420.001

## Introduction

Mutations in IDH1 and IDH2 are found in gliomas and in acute myeloid leukemia. All mutations are heterozygous and result in changes to one of two amino acids: arginine 132 in IDH1, or either arginine 172 or arginine 140 in IHD2. Wild-type IDH1 catalyzes the conversion of isocitrate to α-ketoglutarate (α-KG). The arginine mutations abolish its normal activity and instead mutant IDH1 and IDH2 reduce α-KG to generate the oncometabolite 2-hydroxyglutarate (2-HG) (Ward et al., 2010), which in turn affects the function of multiple α-KG dependent dioxygenases, including the TET family of 5-methylcytosine (5 mC) hydroxylases (Kinney and Pradhan, 2012; McKenney and Levine, 2013). In their *Cancer Cell* 2011 paper, Xu and colleagues examined the effects of excess production of 2-HG on downstream processes that could affect cancer progression. They showed that 2-HG could act as a competitive inhibitor for α-KG-dependent DNA demethylases, specifically Tet2. Ectopic expression of the mutant forms of IDH1 and IDH2 inhibited histone demethylation and 5mC hydroxylation. Examination of glioma samples from patients also showed that mutations in IDH1 were associated with increased histone methylation and decreased 5-hydroxymethylcytosine (5hmC) levels (Xu et al., 2011).

In Supplemental Figure 3I, Xu and colleagues demonstrated that transfection of U-87 MG cells with the mutant IDH1^R132H^ increased the amount of 2-HG in the cells, as compared to transfection with wild-type IDH1 (Xu et al., 2011). This is evidence that mutant IDH1 changes the physiological levels of 2-HG, and is replicated in Protocol 1.

Xu and colleagues first showed that 2-HG can occupy the same binding pocket as α-KG in *Caenorhabditis elegans* KDM7A, indicating it acts as a competitive inhibitor of α-KG. Importantly, they also presented evidence that 2-HG may outcompete α-KG, since 2-HG levels affected many enzymatic functions normally dependent on α-KG. In Figure 3A, they treated U-87 MG cells with cell permeable versions of α-KG and 2-HG, and examined levels of histone methylation by Western Blot. Treatment with increasing amounts of 2-HG led to increases in H3K9me2 and H3K79me2, consistent with the idea that 2-HG inhibited histone demethylases. This effect was abolished by co-treatment with α-KG, confirming a competitive relationship between the two metabolites (Xu et al., 2011). This experiment is replicated in Protocol 2. Xu and colleagues also examined the effect of 2-HG on the TET family of 5 mC hydroxylases using an in vitro system of purified TET2 and double-stranded oligos containing a 5mC restriction digestion site in Figure 8A. Adding increasing concentrations of 2-HG abolished the ability of TET2 to convert 5 mC to 5hmC (Xu et al., 2011). This experiment will be replicated in Protocol 5.

In addition to demonstrating that the metabolite 2-HG can affect the activity of α-KG-dependent enzymes, Xu and colleagues showed that treatment with mutant forms of IDH1 and IDH2 resulted in similar outcomes. In Figure 3D, they transfected U-87 MG cells with IDH1^R132H^ and assessed levels of histone methylation by Western blot. Transfection with IDH1^R132H^ increased histone methylation, and treatment with α-KG abolished this increase in histone methylation, consistent with the idea that α-KG and 2-HG are competitive metabolites (Xu et al., 2011). This experiment will be replicated in Protocol 3. In Figure 7B, they also examined TET activity in the presence of mutant IDH1. While 5hmC levels are normally undetectable in HEK293 cells, transfection with TET catalytic domain (CD)-expressing plasmids increased 5hmC levels to detectable amounts. Co-transfection of TET-CD and wild-type IDH1 or IDH2 increased levels of 5hmC, as expected, while co-transfection of TET-CD with mutant forms of IDH1 and IDH2 decreased 5hmC levels (Xu et al., 2011). This experiment is replicated in Protocol 4.

The work of Xu and colleagues (Xu et al., 2011), along with work from Figueroa and colleagues (Figueroa et al., 2010) and Lu and colleagues (Lu et al., 2012), has generated much interest in the role of altered metabolites in the changing methylation patterns seen in various types of cancer. Using a different cell line than Xu and colleagues, Lu and colleagues demonstrated that mutations in IDH2, similar to mutations in IDH1, also generated abnormal levels of 2-HG which correlated with increased global methylation levels (Lu et al., 2012). Kernystsky and colleagues, Duncan and colleagues and Turcan and colleague have also shown that expression of exogenous mutated IDH genes in immortalized human cancer cell lines or in erythroid progenitor cells caused increased production of 2HG and increased levels of methylation (Duncan et al., 2012; Turcan et al., 2012; Kernytsky et al., 2015). Sasaki and colleagues extended these inquiries by generating conditional knock-in IDH1 mutant mice. These mice displayed elevated serum levels of 2HG and similar patterns of hypermethylation as observed in AML patients (Sasaki et al., 2012). Akbay and colleagues generated IDH2 mutant mice and also observed an increase in global methylation in heart tissue. They also demonstrated that mice carrying IDH mutant xenograft tumors displayed higher serum levels of 2HG (Akbay et al., 2014). Recently, 2-HG production has also been associated with MYC activation in some breast cancers, which also displayed increased levels of methylation as compared to tumors with lower levels of 2-HG (Terunuma et al., 2013).

## Materials and methods

Unless otherwise noted, all protocol information was derived from the original paper, references from the original paper, or information obtained directly from the authors. An asterisk (*) indicates data or information provided by the Reproducibility Project: Cancer Biology core team. A hashtag (#) indicates information provided by the replicating lab.

### Protocol 1: Gas chromatography-mass spectrometry measurement of cellular α-KG and 2-HG concentrations in U87MG cells ectopically expressing mutant IDH1

This protocol describes how to transfect cells with exogenous wild-type IDH1 or mutant IDH1^R132H^ and assess levels of α-KH and 2-HG by gas chromatography-mass spectrometry (GC-MS), as seen in Supplemental Figure 3I.

#### Sampling

- This experiment will be repeated independently 5 times for a final power of at least 92%.
  ○ See Power calculations for details.
- Each experiment consists of three cohorts:
  ○ Cohort 1: U-87 MG cells transfected with vector alone.
  ○ Cohort 1: U-87 MG cells transfected with wild-type IDH1.
  ○ Cohort 1: U-87 MG cells transfected with mutant IDH1.
  Each cohort will be assessed via GC–MS for:
    ■ α-KG levels.
    ■ 2-HG levels.

#### Materials and reagents

**Table:** 

Reagent	Type	Manufacturer	Catalog #	Comments
U-87 MG cells	Cells	ATCC	HTB-14	
N-methyl-N-[tert-butyldimethylsilyl] trifluoroacetamide	Chemical	Sigma–Aldrich	375934-10× 1 M	
Agilent 6890-5973 gas chromatograph-mass spectrometer	Instrument	Agilent	6890-5973	
HP-5MS column	Material	Agilent	19091S-433I	
60 mm tissue culture dishes	Material	Corning	430166	
DMEM; high glucose	Medium	Sigma–Aldrich	D5671	Original unspecified
Opti-MEM Reduced Serum Medium	Medium	Life Technologies	31985-062	Original unspecified
Vector only plasmid (GFP)	Plasmid	Provided by the original authors		
IDH1-IRES-GFP vector	Plasmid	Provided by the original authors		
IDH1^R132H^-IRES-GFP vector	Plasmid	Provided by the original authors		
FBS	Reagent	Sigma–Aldrich	F2442	Original unspecified
Trypsin-EDTA solution, 1×	Reagent	ATCC	ATCC-30-2101	
Penicillin-streptomycin solution	Reagent	ATCC	ATCC-30-2300	
*Trans*IT^®^-LT1 transfection reagent	Reagent	Mirus Bio	MIR 2300	Replaces SunBio-EZ (SunBio)
Methoxyamine hydrochloride	Reagent	Sigma–Aldrich	226904	
Pyridine	Reagent	Sigma–Aldrich	33,553	
GenElute Endotoxin-free Plasmid Maxiprep Kit	Kit	Sigma–Aldrich	PLEX15-1KT	
α-KG	Chemical	Sigma–Aldrich	75892	
L-2-HG	Chemical	Sigma–Aldrich	90790	
0.2 µm filter vials	Material	Restek	25893	
Centrivap	Equipment	Labonco		
Anti-GAPDH-HRP	Antibody	Abcam	ab9385	
Mouse monoclonal IgG_1_ α IDH1	Antibody	Abcam	ab117976	The original catalog number was not specified

#### Procedure

##### Notes

- U-87 MG cells are maintained in DMEM supplemented with 10% FBS at 37°C/5% CO_2_.
- All cells will be sent for mycoplasma testing and STR profiling.

1. Transform, grow up and maxiprep vector only (GFP), IDH1-IRES-GFP, and IDH1^R132H^-IRES-GFP plasmids using a Endo-free Maxiprep kit following manufacturer's instructions.
  a. Confirm plasmid identity by sequencing.
2. Plate U-87 MG cells in 60 mm dishes.
  a. First, run an optimization to determine the growth rate of the cells and optimal number of cells per plate for transfection.
3. 24 hr after plating, transfect cells with plasmids using *Trans*IT-LT1 transfection reagent and Opti-MEM medium according to the manufacturer's protocol.
  a. Transfect 8 µg of DNA per construct using appropriate amount of transfection reagent.
    i. Vector only (GFP).
    ii. Wild-type IDH1 (IDH1-IRES-GFP).
    iii. Mutant IDH1 (IDH1^R132H^-IRES-GFP).
  b. Prepare two plates per cohort; one will be harvested for Western blot confirmation of protein expression (Step 4), the other will be used for metabolite analyses (Step 5).
4. For Western blot: 48 hr after transfection, confirm protein expression by Western blot.
  Note: perform each time cells are transfected.
  a. Run Western blot as outlined in Protocol 2 Steps 3 through 17 with the following modifications:
    i. Blots do not need to be stripped and re-probed.
    ii. Blots will be probed with:
      1. Anti-IDH1; diluted according to the manufacturer's recommendation.
      2. Anti-GAPDH-HRP; 1:5000.
        a. Loading control.
5. For metabolite analysis: 24 hr after transfection, remove culture medium, wash cells with cold PBS and immediately add 10 ml of pre-chilled (−80°C) 80% (vol/vol) methanol. Harvest cells by scraping and lyophilize following the manufacturer's instructions.
  a. Samples will be lyophilized in a speedvac with no heating to keep samples frozen throughout. Immediately after drying remove samples from the speedvac for derivitization.
6. Oximate lyophilized samples with 20 µl 20 mg/ml methoxyamine hydrochloride in pyridine at 30°C for 60 min.
7. Derivatize samples for 30 min at 70°C in 80 μl pyridine and 20 μl N-methyl-N-[tert-butyldimethylsilyl] trifluoroacetamide.
8. Filter samples using 0.2 µm filter vials (PTFE).
9. Inject 3 µl of samples for gas chromatography-mass spectrometry analysis (GC–MS) into Agilent 6890-5973 GC–MS. Use a HP-5MS column (30 m 0.25 mm 0.25 μm) for analysis. Program GC oven temperature from 60°C to 180°C at 5°C/min and from 180°C to 260°C at 10°C/min. Set the flow rate of carrier gas at 1 ml/min. Operate the mass spectrometer in the electron impact (EI) mode at 70 eV.
10. Calculate relative α-KG and 2-HG concentrations by normalizing α-KG (29.86 min) and 2-HG (30.10 min) peak areas to the average of L-threonine (29.58 min), L-serine (29.96 min) and L-phenylalanine (30.74 min) peak areas.
11. Repeat independently four additional times.

#### Deliverables

- Data to be collected:
  ○ Chromatograms and sequence files confirming plasmid identity.
  ○ Data generated determining growth optimization.
  ○ Full image of Western blot showing protein expression and loading controls.
  ○ Mass spectra readouts of all samples.
  ○ Raw values of peak areas for α-KG (29.86 min), 2-HG (30.10 min), L-threonine (29.58 min), L-serine (29.96 min) and L-phenylalanine (30.74 min).
  ○ Quantification of average of peak areas for L-threonine (29.58 min), L-serine (29.96 min) and L-phenylalanine (30.74 min).
  ○ Quantification of relative α-KG and 2-HG concentrations by normalization to average peak areas for L-threonine (29.58 min), L-serine (29.96 min) and L-phenylalanine (30.74 min).
  ○ Bar graphs of relative α-KG or 2-HG concentrations (in percent) for each cell line (as in Supplemental Figure 3I).

#### Confirmatory analysis plan

- Statistical analysis of the replication data:
  ○ Note: At the time of analysis we will perform the Shapiro–Wilk test and generate a quantile–quantile plot to assess the normality of the data. We will also perform Levene's test to assess homoscedasticity. If the data appears skewed we will perform the appropriate transformation in order to proceed with the proposed statistical analysis. If this is not possible we will perform the equivalent non-parametric test.
  ○ One-way MANOVA of α-KG and 2-HG levels in vector-transfected, IDH1-wildtype transfected, and IDH1^R132H^-transfected cells with the following Bonferroni corrected comparisons:
    ■ α-KG levels planned comparisons:
      - vector vs IDH1^WT^.
      - vector vs IDH^R132H^.
      - IDH1^WT^ vs IDH^R132H^.
    ■ 2-HG levels planned comparisons:
      - vector vs IDH1^WT^.
      - vector vs IDH^R132H^.
      - IDH1^WT^ vs IDH^R132H^.
- Meta-analysis of original and replication attempt effect sizes:
  ○ Compute the effect sizes of each comparison, compare them against the reported effect size in the original paper and use a meta-analytic approach to combine the original and replication effects, which will be presented as a forest plot.

#### Known differences from the original study

- Aspects of the Western blot protocol are provided by the replicating lab; complete details of the original protocol were unavailable.
- Since the cell density during transfection is unknown in the original paper, the replicating lab will optimize growth conditions and cell density for transfection.

#### Provisions for quality control

All data obtained from the experiment—raw data, data analysis, control data and quality control data—will be made publicly available, either in the published manuscript or as an open access dataset available on the Open Science Framework (https://osf.io/kvshc/).

- Sequence data confirming plasmid identity.
- Western blots confirming exogenous protein expression.
- STR profiling confirming cell line authenticity.
- Mycoplasma testing confirming lack of contamination.
- Growth characteristics of the cells will be optimized.

### Protocol 2: Western blot to assess histone methylation in U-87 MG cells following treatment with oct-2-HG and/or oct-α-KG

This protocol describes how to treat U-87 MG cells with cell permeable versions of 2-HG and α-KG and assess histone methylation via Western blot, as seen in Figure 3A and Supplemental Figure 3F.

#### Sampling

- The experiment will be repeated independently 3 times for a final power of 84%.
  ○ See Power calculations for details.
- Each experiment consists of four cohorts:
  ○ Cohort 1: untreated U-87 MG cells.
  ○ Cohort 2: U-87 MG cells treated with 10 mM racemic Oct-2-HG.
  ○ Cohort 3: U-87 MG cells treated with 20 mM racemic Oct-2-HG.
  ○ Cohort 4: U-87 MG cells treated with 20 mM racemic Oct-2-HG and 5 mM oct-α-KG.
  Each sample will be blotted for:
    ■ H3K9me2.
    ■ H3K79me2.
    ■ H3.

#### Materials and reagents

**Table:** 

Reagent	Type	Manufacturer	Catalog #	Comments
Mouse monoclonal anti-H3K9me2	Antibody	Abcam	Ab1220	The original catalog number was not specified
Mouse monoclonal anti -H3K79me2	Antibody	Abcam	Ab3594	The original catalog number was not specified
Mouse monoclonal anti -H3	Antibody	Abcam	ab10799	The original catalog number was not specified
Goat Anti-Mouse IgG H&L (HRP)	Antibody	Abcam	ab97023	We will use this for all mouse primaries
U-87 MG cells	Cells	ATCC	HTB-14	
60 mm tissue culture dishes	Material	Corning	430166	
DMEM; high glucose	Medium	Sigma–Aldrich	D5671	Original unspecified
FBS	Reagent	Sigma–Aldrich	F2442	Original unspecified
Oct-α-KG	Reagent	Cayman Chemical	11970	
2S(L)-Oct-2-HG	Reagent	TRC	H942596	Original synthesized in house
2R(L)-Oct-2-HG	Reagent	TRC	H942595	
Protease inhibitor cocktail (mammalian)	Reagent	Sigma–Aldrich	P8340-1ML	Original not specified
TruPAGE TEA-tricine SDS running buffer (20×)	Reagent	Sigma–Aldrich	PCG3001-500 ML	Original not specified
TruPAGE LDS sample buffer (4×)	Reagent	Sigma–Aldrich	PCG3009-10 ML	Original not specified
TruPAGE DTT sample reducer (10×)	Reagent	Sigma–Aldrich	PCG3005-1ML	Original not specified
TruPAGE transfer buffer (20×)	Reagent	Sigma–Aldrich	PCG3011-500 ML	Original not specified
PBS, without MgCl_2_ and CaCl_2_	Reagent	Sigma–Aldrich	D8537	Original not specified
Hybond ECL nitrocellulose membranes; 20 cm × 20 cm	Reagent	GE Healthcare (Sigma–Aldrich)	GERPN2020D	Original not specified
Ponceau S solution; 0.1% (wt/vol) in 5% acetic acid	Reagent	Sigma–Aldrich	P7170	Original not specified
Tris Buffered Saline (TBS); 10× solution	Reagent	Sigma–Aldrich	T5912	Original not specified
Bradford reagent	Reagent	Sigma–Aldrich	B6916	Original not specified
ECL DualVue Western Blotting Markers	Reagent	GE Healthcare (Sigma–Aldrich)	GERPN810	Original not specified
ECL Prime Western blotting system	Reagent	GE Healthcare (Sigma–Aldrich)	GERPN2232	Original not specified
ImageQuant	Software	Molecular Dynamics	Version 5.2	
Typhoon scanner	Equipment	GE Healthcare		

#### Procedure

##### Notes

- U-87 MG cells are maintained in DMEM supplemented with 10% FBS at 37°C/5% CO_2_.
- All cells will be sent for mycoplasma testing and STR profiling.

1. Plate U-87 MG cells in 60 mm dishes.
2. 24 hr after plating, treat cells with 10 or 20 mM racemic Oct-2-HG or 5 mM Oct-α-KG or vehicle (DMSO) for 4–6 hr.
  a. To form racemic mixtures of Oct-2-HG, mix equal amounts of the L and R enantiomers.
3. Wash cells once with cold PBS, then lyse cells in 0.5 mL of SDS loading buffer.
  a. 4× SDS-PAGE loading buffer: 50 mM Tris pH 6.8, 2% SDS, 10% glycerol, 1% B-ME, 12.5 mM EDTA, 0.02% bromophenol blue.
  b. ^#^Measure protein concentration using a CBX assay.
4. Heat lysates at 99°C for 10 min.
5. Run equal amounts of protein per well on a 4–20% SDS-PAGE gel at 220V until ladder marker reaches the bottom of the gel.
6. ^#^Equilibrate gel in transfer buffer for 15 min.
7. ^#^Meanwhile, cut membrane and 4 pieces 3 MM filter paper to size of gel.
  a. Soak membrane in MeOH for a few seconds, then wash with H_2_O.
  b. Soak membrane, 3 MM filter paper and pads in transfer buffer.
    i. Transfer buffer: 38 mM glycine, 47 mM Tris, 11 mM SDS, 20% MeOH.
8. ^#^Assemble transfer cassette:
  a. red pole (+) < clear plate < pad < 2 × 3 MM filter paper < membrane < gel < 2 × 3 MM filter paper < pad < black pole (−).
9. ^#^Add stirring bar and ice box to transfer box and fill box with transfer buffer until cassette is submerged.
  a. Run at 100 V for 1 hr.
10. ^#^Wash membrane in wash buffer for 2 × 5 min.
  a. Wash buffer: 1× PBS with 0.05% Tween-20 and 0.1% sodium azide.
11. ^#^Incubate membrane in blocking buffer for 30 min.
  a. Blocking buffer: 3% non-fat milk in PBS.
12. ^#^Incubate membrane with one of the following primary antibody in blocking buffer for 2 hr at RT or O/N at 4°C (use manufacturer's suggested dilution in blocking buffer).
  a. H3K9me2.
  b. H3K79me2.
  c. H3.
    i. See Step 17 to strip and re-probe the blot with subsequent antibodies.
13. ^#^Wash 5 min 2× with wash buffer.
14. ^#^Incubate membrane with secondary antibody for 90 min at RT (use manufacturer's suggested dilution in blocking buffer).
  a. HRP-conjugated Goat Anti-Mouse IgG H&L: 1: 2000.
15. ^#^Wash 3 × 5 min in wash buffer.
16. ^#^Detect HRP-conjugated secondary antibodies with chemiluminescent detection according to the manufacturer's protocol and image on the Typhoon scanner.
17. Strip the blot in between probes:
  a. Wash the membrane with 100 ml stripping buffer (100 mM beta-mercaptoethanol, 1% SDS 25 mM glycine pH 2.0) for 30 min with agitation.
  b. Wash the stripped membrane twice with Western blotting wash buffer, 600 ml each wash, for 10 min with agitation.
  c. Go to the blocking step of the western blot protocol.
  d. Check that stripping was successful by repeating the detection step (without re-probing). Record image of the stripped gel. This will confirm the first antibody-HRP conjugate is removed and/or inactivated. If the stripping procedure is successful, wash the membrane with washing buffer and repeat the blocking-probing and detection steps for the second antibody.
    i. Note: if stripping is unsuccessful, individual blots will be performed.
18. Quantify intensity of bands on western blots using ImageQuant 5.2. Normalize H3k9me2 and H3K79me2 values to total H3 protein level.
19. Repeat independently 2 additional times.

#### Deliverables

- Data to be collected:
  ○ Full scans of western blots for H3K9me2, H3K79me2 and H3 including ladder.
  ○ Raw values of intensity of western blot bands.
  ○ Quantification of H3K9me2 or H3K79me2 values normalized to total protein level. Levels of H3K9me2 and H3K79me2 in vehicle treated cells are set to relative intensity = 1 and all other conditions are expressed as fold change relative to the values for vehicle treated cells.
  ○ Quantification of average values and standard deviations for each condition for triplicate experiments.
  ○ Bar graph of average ± standard deviation of H3K9me2 and H3K79me2 levels normalized to H3 for each condition. Fold change in intensity relative to vehicle treated cells is plotted on the y axis (as seen in Supp. Figure 3F).

#### Confirmatory analysis plan

- Statistical analysis of the replication data:
  ○ Note: At the time of analysis we will perform the Shapiro–Wilk test and generate a quantile–quantile plot to assess the normality of the data. We will also perform Levene's test to assess homoscedasticity. If the data appears skewed we will perform the appropriate transformation in order to proceed with the proposed statistical analysis. If this is not possible we will perform the equivalent non-parametric test.
  ○ One-way MANOVA of normalized H3K9me2 and H3K79me3 levels in U-87 MG cells untreated or treated with 10 mM Oct-2-HG, 20 mM Oct-2-HG, or 20 mM Oct-2-HG and 5 mM alpha-KG with the following Bonferroni corrected comparisons:
    ■ H3K9me2 planned comparisons:
      - 0 mM 2-HG vs 10 mM 2-HG.
      - 0 mM 2-HG vs 20 mM 2-HG.
      - 20 mM 2-HG vs 5 mM α-KG + 20 mM 2-HG.
    ■ H3K79me3 planned comparisons:
      - 0 mM 2-HG vs 10 mM 2-HG.
      - 0 mM 2-HG vs 20 mM 2-HG.
      - 20 mM 2-HG vs 5 mM α-KG + 20 mM 2-HG.
- Additional statistical analysis for comparison to the original reported data:
  ○ Bonferroni corrected one-sample *t*-tests of normalized H3K9me2 levels of the following conditions compared to 1 (0 mM 2-HG):
    ■ 10 mM 2-HG.
    ■ 20 mM 2-HG.
  ○ Bonferroni corrected one-sample *t*-tests of normalized H3K79me3 levels of the following conditions compared to constant (0 mM 2-HG set to 1):
    ■ 10 mM 2-HG.
    ■ 20 mM 2-HG.
- Meta-analysis of original and replication attempt effect sizes:
  ○ Compute the effect sizes of each comparison, compare them against the reported effect size in the original paper and use a meta-analytic approach to combine the original and replication effects, which will be presented as a forest plot.

#### Known differences from the original study

- The original racemic mixture of Oct-2-HG was synthesized in house by the original lab. The replicating lab is purchasing both L and R enantiomers and mixing them in equal amounts to form a racemic mixture.
- Aspects of the Western blot protocol are provided by the replicating lab; complete details of the original protocol were unavailable.

#### Provisions for quality control

All data obtained from the experiment—raw data, data analysis, control data and quality control data—will be made publicly available, either in the published manuscript or as an open access dataset available on the Open Science Framework (https://osf.io/kvshc/).

- STR profiling confirming cell line authenticity.
- Mycoplasma testing confirming lack of contamination.
- Images of stripped gel membranes confirming stripping was successful.

### Protocol 3: Transfection of U-87 MG cells and determination of histone methylation by western blot

This protocol describes the transfection of U-87 MG cells with the mutant form of IDH1 and assessing methylation by Western blot, as seen in Figure 3D and Supplemental Figure 3J.

#### Sampling

- This experiment will be repeated independently 6 times for a final power of 94%.
  ○ See Power calculations for details.
- Each experiment consists of 5 cohorts:
  ○ Cohort 1: untransfected cells [additional control].
  ○ Cohort 2: Vector transfected cells [additional control].
  ○ Cohort 3: Vector transfected cells + vehicle.
  ○ Cohort 4: IDH1^R132H^ transfected cells + vehicle.
  ○ Cohort 5: IDH1^R132H^ transfected cells + 5 mM oct-α-KG.
  ○ Each cohort is probed with antibodies against:
    ■ H3.
    ■ IDH1.
    ■ H3K4me1.
    ■ H3K4me3.
    ■ H3K9me2.
    ■ H3K27me2.

#### Materials and reagents

**Table:** 

Reagent	Type	Manufacturer	Catalog #	Comments
Mouse monoclonal IgG_3_ α H3	Antibody	Abcam	ab10799	The original catalog number was not specified
Mouse monoclonal IgG_1_ α IDH1	Antibody	Abcam	ab117976	The original catalog number was not specified
Rabbit α H3K4me1	Antibody	Abcam	ab8895	The original catalog number was not specified
Mouse monoclonal IgG_2b_ α H3K4me3	Antibody	Abcam	ab6000	The original catalog number was not specified
Mouse monoclonal IgG_2a_ α H3K9me2	Antibody	Abcam	ab1220	The original catalog number was not specified
Rabbit α H3K27me2	Antibody	Abcam	ab24684	The original catalog number was not specified
Rabbit α H3K79me2	Antibody	Abcam	ab3594	The original catalog number was not specified
U-87 MG cells	Cells	ATCC	HTB-14	
60 mm tissue culture dishes	Material	Corning	430166	Or equivalent
DMEM; high glucose	Medium	Sigma–Aldrich	D5671	Original unspecified
FBS	Reagent	Sigma–Aldrich	F2442	Original unspecified
Empty vector plasmid	Plasmid	Provided by original authors		
IDH1^R132H^ expression vector	Plasmid	Provided by original authors		
*Trans*IT^®^-LT1 transfection reagent	Reagent	Mirus Bio	MIR 2300	Replaces SunBio-EZ (SunBio)
Oct-α-KG	Reagent	Cayman Chemical	11970	
Typhoon scanner	Equipment	GE Healthcare		
ImageQuant	Software	Molecular Dynamics	Version 5.2	
Protease inhibitor cocktail (mammalian)	Reagent	Sigma–Aldrich	P8340-1ML	Original not specified
TruPAGE TEA-tricine SDS running buffer (20×)	Reagent	Sigma–Aldrich	PCG3001-500 ML	Original not specified
TruPAGE LDS sample buffer (4×)	Reagent	Sigma–Aldrich	PCG3009-10 ML	Original not specified
TruPAGE DTT sample reducer (10×)	Reagent	Sigma–Aldrich	PCG3005-1ML	Original not specified
TruPAGE transfer buffer (20×)	Reagent	Sigma–Aldrich	PCG3011-500 ML	Original not specified
PBS, without MgCl_2_ and CaCl_2_	Reagent	Sigma–Aldrich	D8537	Original not specified
Hybond ECL nitrocellulose membranes; 20 cm × 20 cm	Reagent	GE Healthcare (Sigma–Aldrich)	GERPN2020D	Original not specified
Ponceau S solution; 0.1% (wt/vol) in 5% acetic acid	Reagent	Sigma–Aldrich	P7170	Original not specified
Tris buffered saline (TBS); 10× solution	Reagent	Sigma–Aldrich	T5912	Original not specified
Bradford reagent	Reagent	Sigma–Aldrich	B6916	Original not specified
ECL DualVue Western blotting markers	Reagent	GE Healthcare (Sigma–Aldrich)	GERPN810	Original not specified
ECL prime Western blotting system	Reagent	GE Healthcare (Sigma–Aldrich)	GERPN2232	Original not specified
Goat Anti-Rabbit IgG H&L (HRP)	Antibody	Abcam	ab97051	
Goat Anti-Mouse IgG H&L (HRP)	Antibody	Abcam	ab97023	

#### Procedure

##### Notes

- U-87 MG cells are maintained in DMEM supplemented with 10% FBS at 37°C/5% CO_2_.
- All cells will be sent for mycoplasma testing and STR profiling.

1. Plate U-87 MG cells in 60 mm dishes.
2. 24 hr after plating, transfect cells with plasmids (maxiprepped in Protocol 1) using *Trans*IT-LT1 Transfection Reagent according to manufacturer's protocol.
  a. ^#^Transfect 8 µg of DNA per construct using appropriate volume of transfection reagent.
    i. Empty vector.
    ii. IDH1^R132H^ vector.
3. 48 hr after transfection, treat cells with vehicle or 5 mM Oct-α-KG for 6 hr.
  a. Vehicle is DMSO.
4. Wash cells once with cold PBS, then lyse cells in 0.5 ml of SDS loading buffer.
  a. ^#^4 SDS-PAGE loading buffer: 50 mM Tris pH 6.8, 2% SDS, 10% glycerol, 1% B-ME, 12.5 mM EDTA, 0.02% bromophenol blue.
5. Heat lysates at 99°C for 10 min.
6. Run SDS-PAGE gel until ladder marker reaches the bottom of the gel.
7. ^#^Equilibrate gel in transfer buffer for 15 min.
8. ^#^Meanwhile, cut membrane and 4 pieces 3 MM filter paper to size of gel.
  a. Soak membrane in MeOH for a few seconds, then wash with H_2_O.
  b. Soak membrane, 3 MM filter paper and pads in transfer buffer.
    i. Transfer buffer: 38 mM glycine, 47 mM Tris, 11 mM SDS, 20% MeOH.
9. ^#^Assemble transfer cassette:
  a. red pole (+) < clear plate < pad < 2 × 3 MM filter paper < membrane < gel < 2 × 3 MM filter paper < pad < black pole (−).
10. ^#^Add stirring bar and ice box to transfer box and fill box with transfer buffer until cassette is submerged.
  a. Run at 100 V for 1 hr.
11. ^#^Wash membrane in wash buffer for 2 × 5 min.
  a. Wash buffer: 1× PBS with 0.05% Tween-20 and 0.1% sodium azide.
12. ^#^Incubate membrane in blocking buffer for 30 min.
  a. Blocking buffer: 3% non-fat milk in PBS.
13. ^#^Incubate membrane with primary antibody in blocking buffer for 2 hr at room temperature (RT) or overnight at 4°C (use manufacturer's suggested dilution in blocking buffer).
  a. H3.
  b. IDH1.
  c. H3K4me1.
  d. H3K4me3.
  e. H3K9me2.
  f. H3K27me2.
  g. H3K79me2.
14. ^#^Wash 5 min 2× with wash buffer.
15. ^#^Incubate membrane with secondary antibody for 90 min at RT (use manufacturer's suggested dilution in blocking buffer).
  a. HRP-conjugated Goat Anti-Mouse IgG H&L: 1:2000.
  b. HRP-conjugated Goat Anti-Rabbit IgG H&L: 1:2000.
16. ^#^Wash 3 × 5 min in wash buffer.
17. ^#^ Detect HRP-conjugated secondary antibodies with chemiluminescent detection according to the manufacturer's protocol and image on the Typhoon scanner.
18. Strip the blot in between probes:
  a. Wash the membrane with 100 ml stripping buffer (100 mM betamercaptoethanol, 1% SDS 25 mM glycine pH 2.0) for 30 min with agitation.
  b. Wash the stripped membrane twice with Western blotting wash buffer, 600 ml each wash, for 10 min with agitation.
  c. Go to the blocking step of the western blot protocol.
  d. Check that stripping was successful by repeating the detection step (without re-probing). Record image of the stripped gel. This will confirm the first antibody-HRP conjugate is removed and/or inactivated. If the stripping procedure is successful, wash the membrane with washing buffer and repeat the blocking-probing and detection steps for the second antibody.
    i. Note: if stripping is unsuccessful, individual blots will be performed.
19. Quantify intensity of bands on western blots using ImageQuant 5.2. Normalize levels of methylated histones to total H3 protein level. Normalize IDH1^R132H^ + vehicle and IDH1^R132H^ + oct-α-KG treated samples to vector + vehicle samples for each normalized methylated histone.
20. Repeat independently 5 additional times.

#### Deliverables

- Data to be collected:
  ○ Full scans of western blots for H3, IDH1, H3K4me1, H3K4me3, H3K9me2, H3K27me2, and H3K79me2 (as seen in Figure 3D) including ladder.
  ○ Raw values of intensity of western blot bands as measured by ImageQuant 5.2 software.
  ○ Quantification of methylated histone values normalized to total protein level.
  ○ Quantification of average values and standard deviations for each condition. Levels of methylated histone in vector control cells are set to 100% and levels of methylated histone for other conditions are relative to vector control.
  ○ Table of average ± standard deviation of methylated histone levels normalized to H3 for each condition and relative to vector control cells (as seen in Supplemental Figure 3J).

#### Confirmatory analysis plan

- Statistical analysis of the replication data:
  ○ Note: At the time of analysis we will perform the Shapiro–Wilk test and generate a quantile–quantile plot to assess the normality of the data. We will also perform Levene's test to assess homoscedasticity. If the data appears skewed we will perform the appropriate transformation in order to proceed with the proposed statistical analysis. If this is not possible we will perform the equivalent non-parametric test.
  ○ One-way MANOVA of normalized H3K4me1, H3K4me3, H3K9me2, H3K27me2, and H3K79me2 levels from IDH1^R132H^ + vehicle and IDH1^R132H^ + oct-α-KG cells with the following Bonferroni corrected comparisons:
    ■ H3K4me1 levels of IDH1^R132H^ vs IDH1^R132H^ + oct-α-KG.
    ■ H3K4me3 levels of IDH1^R132H^ vs IDH1^R132H^ + oct-α-KG.
    ■ H3K9me2 levels of IDH1^R132H^ vs IDH1^R132H^ + oct-α-KG.
    ■ H3K27me2 levels of IDH1^R132H^ vs IDH1^R132H^ + oct-α-KG.
    ■ H3K79me2 levels of IDH1^R132H^ vs IDH1^R132H^ + oct-α-KG.
  ○ Bonferroni corrected one-sample *t*-tests (outside the MANOVA framework) of normalized levels from IDH1^R132H^ + vehicle of the following conditions compared to constant (vector + vehicle set to 100):
    ○ H3K4me1.
    ○ H3K4me3.
    ○ H3K9me2.
    ○ H3K27me2.
    ○ H3K79me2.
- Meta-analysis of original and replication attempt effect sizes:
  ○ Compute the effect sizes of each comparison, compare them against the reported effect size in the original paper and use a meta-analytic approach to combine the original and replication effects, which will be presented as a forest plot.

#### Known differences from the original study

- While the manufacturer was specified for antibodies used, the exact catalog number was not. The RP:CB core team chose the most appropriate antibody from the manufacturer based on manufacturer's recommended applications and user reviews of the antibody.
- Aspects of the Western blot protocol are provided by the replicating lab; complete details of the original protocol were unavailable.

#### Provisions for quality control

All data obtained from the experiment—raw data, data analysis, control data and quality control data—will be made publicly available, either in the published manuscript or as an open access dataset available on the Open Science Framework (https://osf.io/kvshc/).

- STR profiling confirming cell line authenticity.
- Mycoplasma testing confirming lack of contamination.
- Images of stripped gel membranes confirming stripping was successful.

### Protocol 4: Dot blot to measure of levels of 5hmC in genomic DNA

This protocol describes how to transfect HEK293 cells with vectors expressing the catalytic domain of TET2 (TET2-CD) and wild-type or mutant forms of IHD1 and IDH2 and then assess genomic DNA hydroxymethylation by dot blot, as seen in Figure 7B and Supplemental Figure 7C.

### Sampling

- This experiment will be conducted independently 4 times for a final power of 96%.
  ○ See Power calculations for details.
- Each experiment consists of 9 cohorts:
  ○ Cohort 1: Untransfected cells [additional control].
  ○ Cohort 2: Vector transfected cells.
  ○ Cohort 3: FLAG-TET2-CD transfected cells.
    ■ The catalytic domain of TET2.
  ○ Cohort 4: FLAG-TET2-CM transfected cells.
    ■ CM: mutant version of the TET2 catalytic domain.
  ○ Cohort 5: FLAG-TET2-CD + FLAG-IDH1 transfected cells.
  ○ Cohort 6: FLAG-TET2-CD + FLAG-IDH1^R132H^ transfected cells.
  ○ Cohort 7: FLAG-TET2-CD + FLAG-IDH2 transfected cells.
  ○ Cohort 8: FLAG-TET2-CD + FLAG-IDH2^R140Q^ transfected cells.
  ○ Cohort 9: FLAG-TET2-CD + FLAG-IDH2^R172K^ transfected cells.
  ○ Each cohort will have gDNA spotted out at 5, 10, 25, 50, 100 and 250 ng and probed with anti-5hmC antibody.

#### Materials and reagents

**Table:** 

Reagent	Type	Manufacturer	Catalog #	Comments
Mouse monoclonal IgG_2a_ α Anti-5hmC	Antibody	Active Motif	40000	Original catalog number unspecified
Mouse monoclonal IgG1 α FLAG	Antibody	Sigma–Aldrich	F3165	Original catalog number unspecified
Goat Anti-Mouse IgG H&L (HRP)	Antibody	Abcam	ab97023	
HEK293 cells	Cells	ATCC	CRL-1573	Original unspecified
Typhoon scanner	Equipment	Amersham/ GE Health Sciences	9410	
Hybond ECL nitrocellulose membranes; 20 cm × 20 cm	Reagent	GE Healthcare (Sigma–Aldrich)	GERPN2020D	Original not specified
DMEM; high glucose	Medium	Sigma–Aldrich	D5671	Original unspecified
FBS	Reagent	Sigma–Aldrich	F2442	Original unspecified
Vector alone	Plasmid	Provided by original authors		
FLAG-TET2-CD	Plasmid	Provided by original authors		
FLAG-TET2-CM	Plasmid	Provided by original authors		
FLAG-IDH1	Plasmid	Provided by original authors		
FLAG-IDH1^R132H^	Plasmid	Provided by original authors		
Flag-IDH2	Plasmid	Provided by original authors		
FLAG-IDH2^R140Q^	Plasmid	Provided by original authors		
FLAG-IDH2^R172K^	Plasmid	Provided by original authors		
*Trans*IT-LT1 transfection reagent	Reagent	Mirus Bio	MIR 2300	Replaces SunBio-EZ (SunBio)
Nonfat-dried milk bovine	Reagent	Sigma–Aldrich	M7409	
ECL prime Western blotting system	Reagent	GE Healthcare (Sigma–Aldrich)	GERPN2232	Original not specified
Image Quant 5.2	Software	GE	Version 5.2	
Protease inhibitor cocktail (mammalian)	Reagent	Sigma–Aldrich	P8340-1ML	Original not specified
TruPAGE TEA-Tricine SDS running buffer (20×)	Reagent	Sigma–Aldrich	PCG3001-500 ML	Original not specified
TruPAGE LDS sample buffer (4×)	Reagent	Sigma–Aldrich	PCG3009-10 ML	Original not specified
TruPAGE DTT sample reducer (10×)	Reagent	Sigma–Aldrich	PCG3005-1ML	Original not specified
TruPAGE transfer buffer (20×)	Reagent	Sigma–Aldrich	PCG3011-500 ML	Original not specified
PBS, without MgCl_2_ and CaCl_2_	Reagent	Sigma–Aldrich	D8537	Original not specified
Ponceau S solution; 0.1% (wt/vol) in 5% acetic acid	Reagent	Sigma–Aldrich	P7170	Original not specified
Tris buffered saline (TBS); 10× solution	Reagent	Sigma–Aldrich	T5912	Original not specified
Bradford reagent	Reagent	Sigma–Aldrich	B6916	Original not specified
QIAamp DNA mini kit	Kit	Qiagen	51304	

#### Procedure

##### Notes

- This protocol contains information from Ito and colleagues (Ito et al., 2010).
- HEK293 cells are maintained in DMEM supplemented with 10% FBS at 37°C/5% CO_2_.
- All cells will be sent for mycoplasma testing and STR profiling.

1. Transform, grow up and maxiprep plasmids using an Endo-free Maxiprep kit following the manufacturer's instructions.
  a. Confirm plasmid identity by sequencing.
2. Plate 6 × 10^5^ − 1.2 × 10^6^ HEK293 cells per 60 mm dish.
3. 24 hr after plating, transfect cells with indicated plasmids.
  a. ^#^Transfect cells with 8 µg of DNA per construct using *Trans*IT-LT1 Transfection Reagent according to manufacturer's protocol^#^.
    i. Cohort 1: Untransfected cells.
    ii. Cohort 2: Vector only.
    iii. Cohort 3: FLAG-TET2-CD.
    iv. Cohort 4: FLAG-TET2-CM.
    v. Cohort 5: FLAG-TET2-CD + FLAG-IDH1.
    vi. Cohort 6: FLAG-TET2-CD + FLAG-IDH1^R132H^.
    vii. Cohort 7: FLAG-TET2-CD + FLAG-IDH2.
    viii. Cohort 8: FLAG-TET2-CD + FLAG-IDH2^R140Q^.
    ix. Cohort 9: FLAG-TET2-CD + FLAG-IDH2^R172K^.
^*^For each cohort, transfect two parallel plates; harvest genomic DNA from one plate (proceed to Step 5) and protein from the second plate (proceed to Step 7).
5. 36–40 hr after transfection, isolate genomic DNA from cells on the first plate using the QIAamp kit according to the manufacturer's instructions.
  a. Determine DNA concentration and purity.
6. Dot blot to assess levels of 5hmC:
  a. Quantify gDNA concentration using a NanoDrop. ^#^Spot genomic DNA onto nitrocellulose membrane using a pipet, then crosslink the DNA to the membrane by UV irradiation for 2 min.
    i. The following amounts of genomic DNA should be spotted: 250 ng, 100 ng, 50 ng, 25 ng, 10 ng, and 5 ng.
  b. Bake nitrocellulose membrane at 80°C for ^#^1 hr.
  c. Block membrane with 5% skim milk in TBS with 0.1% Tween 20 (TBST) for 1 hr.
  d. Perform western blot on spotted nitrocellulose with the following antibody: anti-5hmC. Incubate membrane with primary antibody diluted 1:10,000 overnight at 4°C.
  e. Wash membrane three times with TBST.
  f. Incubate membrane with secondary antibody (HRP-conjugated anti-rabbit IgG) diluted 1:2000 for 1 hr at room temperature.
  g. Wash membrane three times with TBST, then treat with ECL and scan with a Typhoon scanner.
  h. Quantify dot-blot using Image-Quanta software.
7. Check expression of exogenous proteins by Western blot using the second plate.
  a. Wash cells once with cold PBS, then lyse cells in 0.5 ml of SDS loading buffer.
    i. ^#^4× SDS-PAGE loading buffer: 50 mM Tris pH 6.8, 2% SDS, 10% glycerol, 1% B-ME, 12.5 mM EDTA, 0.02% bromophenol blue.
  b. Heat lysates at 99°C for 10 min.
  c. Run SDS-PAGE gel until ladder marker reaches the bottom of the gel.
  d. ^#^Equilibrate gel in transfer buffer for 15 min.
  e. ^#^Meanwhile, cut membrane and 4 pieces 3 MM filter paper to size of gel.
    i. Soak membrane in MeOH for a few seconds, then wash with H_2_O.
    ii. Soak membrane, 3 mM filter paper and pads in transfer buffer.
    iii. Transfer buffer: 38 mM glycine, 47 mM Tris, 11 mM SDS, 20% MeOH
  f. ^#^Assemble transfer cassette:
    i. red pole (+) < clear plate < pad < 2 × 3 MM filter paper < membrane < gel < 2 × 3 MM filter paper < pad < black pole (−).
  g. ^#^Add stirring bar and ice box to transfer box and fill box with transfer buffer until cassette is submerged.
    i. Run at 100 V for 1 hr.
  h. ^#^Wash membrane in wash buffer for 2 × 5 min.
    i. Wash buffer: 1× PBS with 0.05% Tween-20 and 0.1% sodium azide.
  i. ^#^Incubate membrane in blocking buffer for 30 min.
    i. Blocking buffer: 3% non-fat milk in PBS.
  j. ^#^Incubate membrane with primary antibody in blocking buffer for 2 hr at RT or O/N at 4°C (use manufacturer's suggested dilution in blocking buffer).
    i. α FLAG.
  k. ^#^Wash 5 min 2× with wash buffer.
  l. ^#^Incubate membrane with secondary antibody for 90 min at RT (use manufacturer's suggested dilution in blocking buffer).
    i. HRP-conjugated Goat Anti-Mouse IgG H&L: 1:2000.
  m. ^#^Wash 3 × 5 min in wash buffer.
  n. ^#^ Detect HRP-conjugated secondary antibodies with chemiluminescent detection according to the manufacturer's protocol and image on the Typhoon scanner.
  o. Quantify intensity of dots on western blots using ImageQuant 5.2.
    i. Normalize values to FLAG-TET2-CD transfected cells.
8. Repeat independently three additional times.

#### Deliverables

- Data to be collected:
  ○ Chromatograms and sequence files confirming plasmid identity.
  ○ DNA concentration and purity data.
  ○ Full scans of dot blots for anti-5hmC and western blots for anti-FLAG (as seen in Figure 7B).
  ○ Raw values of intensity of dot blot as measured by Image-Quanta software.
  ○ Quantification of 5hmc values relative to TET2-CD.
  ○ Quantification of average values and standard deviations for each condition for all experiments.
  ○ Bar graph and table of average values and standard deviations relative to TET2-CD samples (as seen in Figure 7B and Supplemental Figure 7C).

#### Confirmatory analysis plan

- Statistical analysis of the replication data:
  ○ Note: At the time of analysis we will perform the Shapiro–Wilk test and generate a quantile–quantile plot to assess the normality of the data. We will also perform Levene's test to assess homoscedasticity. If the data appears skewed we will perform the appropriate transformation in order to proceed with the proposed statistical analysis. If this is not possible we will perform the equivalent non-parametric test.
  ○ Comparison of the various genotypes for each of the DNA concentrations.
    ■ Bonferonni corrected one-sample *t*-test of normalized 5hmC levels of the following cohorts compared to constant (TET2-CD set to 1):
      - TET-2CD + IDH1.
      - TET-2CD + IDH1^R132H^.
      - TET-2CD + IDH2.
      - TET-2CD + IDH2^R140Q^.
      - TET-2CD + IDH2^R172K^.
- Meta-analysis of original and replication attempt effect sizes:
  ○ Compute the effect sizes of each comparison, compare them against the reported effect size in the original paper and use a meta-analytic approach to combine the original and replication effects, which will be presented as a forest plot.

#### Known differences from the original study

- Aspects of the Western blot protocol are provided by the replicating lab; complete details of the original protocol were unavailable.

#### Provisions for quality control

All data obtained from the experiment—raw data, data analysis, control data and quality control data—will be made publicly available, either in the published manuscript or as an open access dataset available on the Open Science Framework (https://osf.io/kvshc/).

- Sequence data confirming plasmid identity.
- Western blots confirming exogenous protein expression.
- STR profiling confirming cell line authenticity.
- Mycoplasma testing confirming lack of contamination.

### Protocol 5: Radiolabeled 5mC-5hmC conversion assay

This protocol describes how to run the in vitro assay to examine the effect of 2-HG on the TET family of methyl hydroxylases, as seen in Figure 8A.

#### Sampling

- This experiment will be performed independently a total of 6 times for a final power of ≥80%.
  ○ The original data is qualitative, thus to determine an appropriate number of replicates to initially perform, sample sizes based on a range of potential variance was determined.
  ○ See Power calculations for details.
- Each experiment consists of 8 cohorts:
  ○ No recombinant protein.
  ○ FLAG-TET2-CD + vehicle.
  ○ FLAG-TET2-CD + 10 mM D-2-HG.
  ○ FLAG-TET2-CD + 25 mM D-2-HG.
  ○ FLAG-TET2-CD + 50 mM D-2-HG.
  ○ FLAG-TET2-CD + 10 mM L-2-HG.
  ○ FLAG-TET2-CD + 25 mM L-2-HG.
  ○ FLAG-TET2-CD + 50 mM L-2-HG.
  ○ Each cohort will detect:
    ■ 5m-dCMP.
    ■ 5hm-dCMP.

#### Materials and reagents

**Table:** 

Reagent	Type	Manufacturer	Catalog #	Comments	
D-2-HG	Reagent	Sigma–Aldrich	H8378		
L-2-HG	Reagent	Sigma–Aldrich	90790		
Sf9 cells	Cells	ATCC	CRL-1711	Original unspecified	
Shrimp alkaline phosphatase	Reagent	New England Biolabs	MO371S		
T4 polynucleotide kinase	Reagent	Sigma–Aldrich	KEM0006		
DNase I	Reagent	Sigma–Aldrich	AMPD1		
Phosphodiesterase I	Reagent	Sigma–Aldrich	P3243		
PEI-cellulose TLC plate	Material	Sigma–Aldrich	Z122882		
FLAG-TET2-CD viral particles	Virus	Provided by the original authors			
Anti-Flag M2 antibody agarose affinity gel	Reagent	Sigma–Aldrich	A2220		
Flag peptide	Reagent	Sigma–Aldrich	F4799		
α-KG	Reagent	Sigma–Aldrich	75,892		
GenElute PCR Clean-Up Kit	Kit	Sigma–Aldrich	NA1020-1KT	Replaces Qiagen cat no. 28304	
[γ-32]ATP	Reagent	Perkin Elmer	BLU502H/NEG502H		
MspI methyltransferase	Reagent	NEB	M0215L		
MspI restriction endonuclease	Reagent	NEB	R0106T		
DNA duplex oligonucleotide substrate	oligo	Integrated DNA Technologies	custom 5′-GTGTTCTTTCAGCTCCGGTCACGCTGACCAGC-3′ as a duplex oligo, HPLC purified at 1 umole scale maybe higher depending on recovery		
M13-F primer	oligo	Integrated DNA Technologies			CCAGTCACGACGTTGTAAAACG
M13-R primer	oligo	Integrated DNA Technologies			CCAGTCACGACGTTGTAAAACG
JumpStart REDTaq DNA Polymerase	Reagent	Sigma	D8189-50UN		
dNTP mix 10 mM	Reagent	Sigma	D7295-.2 ML		
BlueView TAE buffer	Buffer	Sigma	T8935-1L		
Molecular biology grade water	Reagent	Sigma	W4502-1L		

#### Procedure

Note: This protocol contains information from Ito and colleagues (2010).

1. Generate recombinant FLAG-TET2-CD virus from supplied virus stock.
  a. ^#^Infect a 5 ml culture with 0.1 ml of virus stock supplied.
    i. Grow in a stationary tissue culture flask at 27°C.
  b. ^#^After 5 days, collect the virus. Simultaneously, start a 50 ml suspension culture at 27°C with 140 rpm shaking.
    i. Confirm viral insert identity by sequencing using M13F and R primers and REDTaq polymerase, followed by gel purification and sequencing of PCR product.
  c. ^#^After culturing for 3 days, infected the suspension culture with 2.5 ml of virus stock.
  d. ^#^4 days after infection collect virus. Simultaneously, start new 50 ml suspension cultures for protein expression.
    e. ^#^After 3 days of culture, the suspension cultures are infected with 2.5 ml virus.
  e. ^#^After 3 days of infection the cells expressing recombinant protein are collected by centrifugation and stored at −80°C until the protein is to be purified.
    i. ^#^More round of expression may be required depending on expression level.
  f. Purify baculovirus expressed recombinant FLAG-TET2-CD from insect Sf9 cells with anti-Flag M2 antibody agarose affinity gel and elute with buffer containing 10 mM Tris–HCl pH 8.0, 150 mM NaCl, 1 mM DTT, 15% glycerol and 0.2 μg/µl Flag peptide.
  g. Note; generate sufficient recombinant protein to use in a total of 6 replicates of this protocol.
2. ^#^Prepare methylated oligonucleotide substrate.
  a. Treat unmethylated DNA duplex oligo with MspI methyltransferase for 2 hr at 37°C following manufacturer's instructions.
  b. Purify with a QiaQuick Nucleotide Removal kit following manufacturer's instructions.
3. Incubate 5 µg of the purified recombinant TET2-CD protein and various concentrations of vehicle only, D-2-HG, or L-2-HG with 0.5 μg methylated oligonucleotide substrate in vehicle (50 mM HEPES (pH 8), 75 μM Fe(NH_4_)_2_(SO_4_)_2_, 2 mM ascorbate) and 0.1 mM α-KG for 3 hr at 37°C.
  a. See cohorts for detailed concentrations to use.
  b. ^#^If necessary, concentrate protein to ensure the final reaction volume is between 100–1000 µl.
  c. Purify oligonucleotide substrates using a GenElute PCR Clean-Up Kit following manufacture's instructions.
4. Digest oligonucleotides with 1 U/μg MspI restriction endonuclease at 37°C for ^#^2 hr following manufacturer's instructions.
5. Treat digested DNA with 1U/μmol shrimp alkaline phosphatase at 37°C for ^#^2 hr.
  a. ^#^Heat inactivate at 65°C for 10 min.
6. Label DNA with [γ-32]ATP and polynucleotide kinase.
  a. ^#^Add 1 µl of [γ-32]ATP at 3000 Ci/mmol, 5 mCi/ml and 1 µl polynucleotide kinase to the previous reaction.
  b. ^#^Incubate for 1 hr at 37°C.
7. Ethanol precipitate labeled fragments.
  i. ^#^Add 3M NaOAc to a final concentration of 0.3M.
  ii. ^#^Add 2 vol 100% EtOH.
  iii. ^#^Incubate mixture at on dry ice for 20 min.
  iv. ^#^Centrifuge in a microfuge at 4°C at maximum speed for 10 min.
  v. ^#^Remove supernatant and air dry pellet.
  vi.^#^Resuspend.
8. Digest labeled fragments with 10 µg DNAse I and 10 μg phosphodiesterase I in the presence of 15 mM MgCl_2_ and 2 mM CaCl_2_ at 37 °C for ^#^2 hr.
9. Spot 1 µl of digestion product from step 8 onto a PEI-cellulose TLC plate and separate in an isobutyric acid/water/ammonium hydroxide (66:20:2) buffer.
10. Dry the TLC plate and then expose to film.
11. Quantify intensity of 5hmC bands.
  a. Normalize values to FLAG-TET2-CD + vehicle.
12. Repeat independently five additional times starting at Step 2.

#### Deliverables

- Data to be collected:
  ○ Sequencing data confirming viral insert identity.
  ○ Data about viral titer and amount of and quality of protein generated.
  ○ Scans of films exposed to TLC plate (as in Figure 8A, left).
  ○ Raw values of intensity of 5hm-dCMP (5hmC) spots.
  ○ Quantification of 5hmC intensity relative to FLAG-TET2-CD (recombinant protein) + vehicle sample.
  ○ Quantification of average values and standard deviations for each condition for triplicate experiments.
  ○ Bar graph of relative 5hmC intensity for each sample with standard deviations (as in Figure 8A, right).

#### Confirmatory analysis plan

- Statistical Analysis of the Replication Data:
  ○ Note: At the time of analysis we will perform the Shapiro–Wilk test and generate a quantile–quantile plot to assess the normality of the data. We will also perform Levene's test to assess homoscedasticity. If the data appears skewed we will perform the appropriate transformation in order to proceed with the proposed statistical analysis. If this is not possible we will perform the equivalent non-parametric test.
  ○ Two-way ANOVA of normalized 5hmC levels of TET2-CD protein treated with D-2-HG or L-2-HG with the following Bonferroni corrected comparisons:
    - 10 mM D-2-HG vs 10 mM L-2-HG.
    - 50 mM D-2-HG vs 50 mM L-2-HG.
    - 10 mM D-2-HG vs 50 mM D-2-HG.
  ○ Bonferroni corrected one-sample *t*-tests (outside the ANOVA framework) of normalized 5hmC levels of TET2-CD protein treated with the following concentrations of D-2-HG compared to constant (TET2-CD + vehicle set to 1):
    - 10 mM D-2-HG.
    - 50 mM D-2-HG.
- Meta-analysis of original and replication attempt effect sizes:
  ○ The replication data (mean and 95% confidence interval) will be plotted with the original reported data value plotted as a single point on the same plot for comparison.

#### Known differences from the original study

- The lab provided the protocol for expansion of the viral aliquot shared by the original authors for generation of the recombinant FLAG-TET2 protein.

#### Provisions for quality control

All data obtained from the experiment—raw data, data analysis, control data and quality control data—will be made publicly available, either in the published manuscript or as an open access dataset available on the Open Science Framework (https://osf.io/kvshc/).

- Sequence data confirming viral insert identity.
- Data about viral titer and amount of and quality of protein generated.

##### Power calculations

Power calculations are performed to calculate the number of samples required to achieve at least 80% power and the indicated alpha error. For a detailed breakdown of all power calculations, please see spreadsheet at https://osf.io/gnsti/wiki/home/.

#### Protocol 1

##### Summary of original data

- Note: Data estimated from published figures.

**Table:** 

Supp. Figure 3I: Levels of α-KG with WT or mutant IDH1	Mean	SD	N
Vector-transfected U-87 MG cells	84	0*	2
WT IDH-transfected U-87 MG cells	120	7.8	2
IDH^R132H^-transfected U-87 MG cells	41	14	2

Supp. Figure 3I: Levels of 2-HG with WT or mutant IDH1.	Mean	SD	N
Vector-transfected U-87 MG cells	90	0*	2
WT IDH-transfected U-87 MG cells	140	0*	2
Mutant IDH-transfected U-87 MG cells	1730	14	2

* Because the original data reported null variances, the calculations below used the average of the non-null variances, 11.9, in place of a SD of 0.

##### Test family

- Due to a lack of raw original data, we are unable to perform power calculations using a MANOVA. We are determining sample size calculations using a two-way ANOVA.
- Two-way ANOVA followed by Bonferroni corrected comparisons.

##### Power calculations

- Calculations were performed with R software, version 3.1.2 (R Core Team, 2014) and G*Power software, version 3.1.7 (Faul et al., 2007).

**Table:** 

ANOVA calculations; α = 0.05				
F(1,6) metabolite	Partial η2	Effect size *f*	Power	Total sample size
6702.3	0.999106	33.43005	99.9%	7*

* With 5 samples per group (30 samples total), power achieved is 99.9%.

**Table:** 

Corrected *t*-test sample size calculations; α = 0.0083333					
	Group 1	Group 2	Effect size *d*	Power	Sample size per group
α-KG	Vector	IDH1-WT	3.57815	80.1%*	4*
	Vector	IDH1-R132H	3.30960	92.0%	5
	IDH1-WT	IDH1-R132H	6.97125	97.4%†	3†
2HG	Vector	IDH1-WT	4.20168	93.1%‡	4‡
	Vector	IDH1-R132H	126.22669	99.9%§	2§
	IDH1-WT	IDH1-R132H	122.37832	99.9%#	2#

* With a sample size of 5 per group, the achieved power is 95.7%.

† With a sample size of 5 per group, the achieved power is 99.9%.

‡ With a sample size of 5 per group, the achieved power is 99.2%.

§ With a sample size of 5 per group, the achieved power is 99.9%.

# With a sample size of 5 per group, the achieved power is 99.9%.

##### Sensitivity calculations

- Comparing 2-HG levels from Vector to IDH1 WT:
  ○ Based on a sample size of 4 per group, we will be able to see an effect size of 3.3710662 with α = 0.01 and a power of 80%.

#### Protocol 2

##### Summary of original data

- Note: Data estimated from published figures.

**Table:** 

Supp. Fig. 3F: Quantification of Figure 3A Western Blots		Mean	SD	N
Untreated cells	H3K9me2/H3 ratio	1	0	3
	H3K79me2/H3 ratio	1	0	3
10 mM oct-2-HG treated cells	H3K9me2/H3 ratio	3.8	0.5	3
	H3K79me2/H3 ratio	8.5	1.5	3
20 mM oct-2-HG treated cells	H3K9me2/H3 ratio	5.5	0.3	3
	H3K79me2/H3 ratio	17.2	2.4	3
20 mM oct-2-HG + 5 mM oct-α-KG treated cells	H3K9me2/H3 ratio	0.6	0.3	3
	H3K79me2/H3 ratio	0.9	0.3	3

##### Test family

- Due to a lack of raw original data, we are unable to perform power calculations using a MANOVA. We are determining sample size calculations using a two-way ANOVA.
- Two-way ANOVA followed by Bonferroni corrected comparisons.

##### Power calculations

- Calculations were performed with R software, version 3.1.2 (R Core Team, 2014) and G*Power software, version 3.1.7 (Faul et al., 2007).

**Table:** 

ANOVA calculations; α = 0.05				
F(1,16) histone	Partial η2	Effect size *f*	A priori power	Total sample size
235.0200	0.936260	3.83259	99.9%*	10*

* With 3 samples per group (12 total), achieved power is 99.9%.

**Table:** 

Corrected *t*-tests sample size calculations; α = 0.0083					
	Group 1	Group 2	Effect size *d*	Power	Sample size per group
H3K9me2	Vehicle treated cells	10 mM Oct-2-HG treated cells	11.05934	99.9%	3
H3K79me2			8.92288	99.9%	3
H3K9me2	Vehicle treated cells	20 mM Oct-2-HG treated cells	23.55408	99.9%	3
H3K79me2			14.24069	99.9%	3
H3K9me2	20 mM Oct-2-HG treated cells	20 mM Oct-2-HG + 5 mM oct-α-KG treated cells	28.82353	99.9%	3
H3K79me2			16.46129	99.9%	3

##### Test family

- This is an additional analysis to allow a direct comparison with the original study.
- Bonferroni corrected one-sample *t*-tests compared to 1 (vehicle treated cells).

##### Power calculations

- Calculations were performed with G*Power software, version 3.1.7 (Faul et al., 2007).

**Table:** 

Bonferroni corrected t-tests; α = 0.0083					
	Group	Constant	Effect size d	A Priori power	Sample size per group
H3K9me2	10 mM Oct-2-HG treated cells	1	9.65517	90.3%	3
H3K79me2			8.62069	84.4%	3
H3K9me2	20 mM Oct-2-HG treated cells	1	26.47059	99.9%	3
H3K79me2			11.65468	o 96.6%	3

#### Protocol 3

##### Summary of original data

- Note: Data estimated from published figure.

**Table:** 

Supp. Figure 3J: quantification of Western blot band intensities from Figure 3D normalized to vector control	Mean	SD	N
With vector + vehicle			
H3K4me1/H3 ratio	100	Unspecified	3
H3K4me3/H3 ratio	100	unspecified	3
H3K9me3/H3 ratio	100	unspecified	3
H3K27me2/H3 ratio	100	unspecified	3
H3K79me2/H3 ratio	100	unspecified	3
With IDH1^R132H^ + vehicle			
H3K4me1/H3 ratio	209	36	3
H3K4me3/H3 ratio	466	64	3
H3K9me3/H3 ratio	283	56	3
H3K27me2/H3 ratio	232	24	3
H3K79me2/H3 ratio	267	47	3
With IDH1^R132H^ and oct-α-KG			
H3K4me1/H3 ratio	105	16	3
H3K4me3/H3 ratio	274	25	3
H3K9me3/H3 ratio	126	21	3
H3K27me2/H3 ratio	99	9	3
H3K79me2/H3 ratio	130	20	3

##### Test family

- Due to a lack of raw original data, we are unable to perform power calculations using a MANOVA. We are determining sample size calculations using a two-way ANOVA.
- Two-way ANOVA followed by Bonferroni corrected comparisons.

##### Power calculations

- Calculations were performed with R software, version 3.1.2 (R Core Team, 2014) and G*Power software, version 3.1.7 (Faul et al., 2007).

**Table:** 

ANOVA calculations; α = 0.05				
F(1,20) cell treatments	Partial η2	effect size *f*	Power	Total Sample size
119.5629	0.85670	2.44502	97.1%*	12*

* With 6 samples per group (for a total of 60 samples), the power achieved is 99.9%.

**Table:** 

Corrected *t*-test sample size calculations; α = 0.005					
Group 1	Group 2	Histone	Effect size *d*	Power	Sample size per group
IDH1^R132H^ + vehicle	IDH1^R132H^ + oct-α-KG	H3K4me1/H3 ratio	3.73338	94.2%*	5*
		H3K4me3/H3 ratio	3.95184	82.1%†	4†
		H3K9me3/H3 ratio	3.71240	93.9%‡	5‡
		H3K27me2/H3 ratio	7.33811	95.0%§	3§
		H3K79me2/H3 ratio	3.79314	94.9%#	5#

* With a sample size of 6 per group, the achieved power is 98.9%.

† With a sample size of 6 per group, the achieved power is 99.5%.

‡ With a sample size of 6 per group, the achieved power is 98.8%.

§ With a sample size of 6 per group, the achieved power is 99.9%.

# With a sample size of 6 per group, the achieved power is 99.1%.

##### Test family

- Outside the ANOVA framework
- Bonferroni corrected one-sample *t*-tests compared to 1 (vector + vehicle).

##### Power calculations

- Calculations were performed with G*Power software, version 3.1.7 (Faul et al., 2007).

**Table:** 

Corrected *t*-test sample size calculations; α = 0.005					
Group 1	Constant	Histone	Effect size *d*	Power	Sample size per group
IDH1^R132H^ + vehicle	100	H3K4me1/H3 ratio	3.02778	94.2%	6
		H3K4me3/H3 ratio	5.71875	92.2%*	4*
		H3K9me3/H3 ratio	3.26786	82.9%†	5†
		H3K27me2/H3 ratio	5.50000	90.2%‡	4‡
		H3K79me2/H3 ratio	3.55319	88.8%§	5§

* With a sample size of 6 per group, the achieved power is 99.9%.

† With a sample size of 6 per group, the achieved power is 96.9%.

‡ With a sample size of 6 per group, the achieved power is 99.9%.

§ With a sample size of 6 per group, the achieved power is 98.7%.

#### Protocol 4

##### Summary of original data

○ Note: Values estimated from published figure.

**Table:** 

Figure 7B: Relative 5hmC intensity	Mean	SD	N
50 ng Genomic DNA			
Vector	0	0.01	3
TET2-CD	1	0	3
TET2-CM	0	0.01	3
TET2-CD + IDH1	2.5	0.3	3
TET2-CD + IDH1^R132H^	0.29	0.1	3
TET2-CD + IDH2	2.6	0.11	3
TET2-CD + IDH2^R40Q^	0.31	0.07	3
TET2-CD + IDH2^R172K^	0.31	0.09	3

##### Test family

- Bonferroni corrected one-sample *t*-tests compared to 1 (TET2-CD).

##### Power calculations

- Power calculations were performed using G*Power software, version 3.1.7 (Faul et al., 2007).

**Table:** 

Corrected *t*-test sample size calculations; α = 0.01				
Group 1: TET2 +	Constant	Effect size *d*	Power	Sample size per group
IDH1	1	5.00000	95.9%	4
IDH1^R132H^	1	7.10000	99.9%	4
IDH2	1	14.54545	99.8%*	3*
IDH2^R140Q^	1	9.85714	94.6%†	3†
IDH2^R172K^	1	7.66667	82.9%‡	3‡

* With a sample size of 4 per group, the achieved power is 99.9%.

† With a sample size of 4 per group, the achieved power is 99.9%.

‡ With a sample size of 4 per group, the achieved power is 99.9%.

#### Protocol 5

##### Summary of original data

- Note: Data estimated from published figures.

**Table:** 

Figure 8A: TLC blot intensities	Mean
TET2 + vehicle	1
TET2 + 10 mM D-2-HG	0.67
TET2 + 25 mM D-2-HG	0.45
TET2 + 50 mM D-2-HG	0.17
TET2 + 10 mM L-2-HG	0.05
TET2 + 25 mM L-2-HG	0.03
TET2 + 50 mM L-2-HG	0.03

##### Test family

- One way ANOVA followed by Bonferroni corrected comparisons.
- Outside the ANOVA framework
  ○ Bonferroni corrected one-sample *t*-tests compared to 1 (TET2 + vehicle).

##### Power calculations

- Because the original data presented does not have variance (s.e.m. or s.d.), we have performed power calculations using several different levels of calculated variance and an assumed number of replicates to determine a suitable number of replications to perform.
- Calculations were performed with R software, version 3.1.2 (R Core Team, 2014) and G*Power software, version 3.1.7 (Faul et al., 2007).

**Table:** 

Calculated variances and assumed N						
Figure 8A: dot blot intensities	Mean	N	2%	15%	28%	40%
TET2 + vehicle	1	3	n/a*	n/a*	n/a*	n/a*
TET2 + 10 mM D-2-HG	0.67	3	0.0134	0.1005	0.1876	0.268
TET2 + 25 mM D-2-HG	0.45	3	0.009	0.0675	0.126	0.18
TET2 + 50 mM D-2-HG	0.17	3	0.0034	0.0255	0.0476	0.068
TET2 + 10 mM L-2-HG	0.05	3	0.001	0.0075	0.014	0.02
TET2 + 25 mM L-2-HG	0.03	3	0.0006	0.0045	0.0084	0.012
TET2 + 50 mM L-2-HG	0.03	3	0.0006	0.0045	0.0084	0.012

* Because each replicate will be normalized to TET2 + vehicle this will not have a variance associated with it. And thus the TET2 + vehicle is also not include in the ANOVA calculation.

#### 2% variance

**Table:** 

ANOVA calculations; α = 0.05				
F(2,12) interaction	Partial η2	Effect size *f*	Power	Total sample size
1910.6	0.99687	17.8434	98.2%*	9*

* With 12 total samples, the power achieved is 99.9%.

**Table:** 

Corrected *t*-test sample size calculations; α = 0.01				
Group 1	Group 2	Effect size *d*	Power	Sample size per group
10 mM D-2-HG	10 mM L-2-HG	65.25231	99.9%	2
50 mM D-2-HG	50 mM L-2-HG	57.34623	99.9%	2
10 mM D-2-HG	50 mM D-2-HG	51.14839	99.9%	2

Corrected *t*-test sample size calculations; α = 0.01				
Group 1:	Constant	Effect size *d*	Power	Sample size per group
10 mM D-2-HG	1	24.62687	97.7%	2
50 mM D-2-HG	1	244.11765	99.9%	2

#### 15% variance

**Table:** 

ANOVA calculations; α = 0.05				
F(2,12) interaction	Partial η2	Effect size *f*	Power	Total sample size
2.37930	0.84987	2.37930	93.8%	12*

* With 12 total samples, the power achieved is 99.9%.

**Table:** 

Corrected *t*-test sample size calculations; α = 0.01				
Group 1	Group 2	Effect size *d*	Power	Sample size per group
10 mM D-2-HG	10 mM L-2-HG	8.70031	99.9%	3
50 mM D-2-HG	50 mM L-2-HG	7.64616	99.4%	3
10 mM D-2-HG	50 mM D-2-HG	6.81978	97.9%	3

Corrected *t*-test sample size calculations; α = 0.01				
Group 1:	Constant	Effect size *d*	Power	Sample size per group
10 mM D-2-HG	1	3.28358	87.2%	6
50 mM D-2-HG	1	32.54902	99.5%	3

#### 28% variance

**Table:** 

ANOVA calculations; α = 0.05				
F(2,12) interaction	Partial η2	Effect size *f*	Power	Total sample size
9.7548	0.61916	1.27507	86.1%	12

Corrected t-test sample size calculations; α = 0.01				
Group 1	Group 2	Effect size *d*	Power	Sample size per group
10 mM D-2-HG	10 mM L-2-HG	4.66088	97.9%	4
50 mM D-2-HG	50 mM L-2-HG	4.09616	93.6%	4
10 mM D-2-HG	50 mM D-2-HG	3.65346	86.6%	4

Corrected *t*-test sample size calculations; α = 0.01				
Group 1:	Constant	Effect size *d*	Power	Sample size per group
10 mM D-2-HG	1	1.75906	80.3%	14
50 mM D-2-HG	1	17.43697	94.0%	4

#### 40% variance

**Table:** 

ANOVA calculations; α = 0.05				
F(2,12) interaction	Partial η2	Effect size *f*	Power	Total sample size
4.7765	0.44323	0.892237	82.2%	17*

* With 18 total samples, the power achieved is 85.3%.

**Table:** 

Corrected *t*-test sample size calculations; α = 0.01				
Group 1	Group 2	Effect size *d*	Power	Sample size per group
10 mM D-2-HG	10 mM L-2-HG	3.26262	92.8%	5
50 mM D-2-HG	50 mM L-2-HG	2.86731	83.9%	5
10 mM D-2-HG	50 mM D-2-HG	2.55742	86.3%	6

Corrected *t*-test sample size calculations; α = 0.01				
Group 1:	Constant	Effect size *d*	Power	Sample size per group
10 mM D-2-HG	1	1.23134	80.8%	27
50 mM D-2-HG	1	12.20588	84.6%	5

In order to produce quantitative replication data, we will run the experiment six times. Each time we will quantify band intensity. We will determine the standard deviation of band intensity across the biological replicates and combine this with the reported value from the original study to simulate the original effect size. We will use this simulated effect size to determine the number of replicates necessary to reach a power of at least 80%. We will then perform additional replicates, if required, to ensure that the experiment has more than 80% power to detect the original effect.
